# Emerging Pharmacological Approaches for the Treatment of Arterial Hypertension

**DOI:** 10.3390/biomedicines13040790

**Published:** 2025-03-25

**Authors:** Francesca Schinzari, Rossella Montenero, Carmine Cardillo, Manfredi Tesauro

**Affiliations:** 1 Department of Aging, Policlinico A. Gemelli IRCCS, 00168 Rome, Italy; francesca.schinzari@policlinicogemelli.it (F.S.); rossellamontenero2@gmail.com (R.M.); 2 Department of Translational Medicine and Surgery, Catholic University, 00168 Rome, Italy; 3 Department of Systems Medicine, Tor Vergata University, 00133 Rome, Italy; mtesauro@tiscali.it

**Keywords:** resistant hypertension, anti-hypertensive drugs, renin–angiotensin–aldosterone system, obesity, clinical trials

## Abstract

Despite the availability of several drug classes for the treatment of hypertension, the current approaches to high blood pressure (BP) are not fully satisfying the needs of this patient population. As a result, in recent years, many clinical trials have investigated novel pharmacological approaches for lowering high BP. As overactivity of the renin–angiotensin–aldosterone system is often present in hypertensive patients, especially those with resistant hypertension, several studies have focused on novel strategies to counteract this phenomenon by the use of non-steroidal inhibitors of the mineralocorticoid receptors, aldosterone synthase inhibitors or RNA-targeting therapies to inhibit the hepatic synthesis of angiotensinogen. The latter approach in particular might offer the additional advantage of reducing the daily pill burden of these patients, hence mitigating the common occurrence of non-adherence to treatment. Because obesity and diabetes are common risk factors for hypertension (a high percentage of individuals with resistant hypertension being obese), numerous investigations have analyzed the BP-lowering effects of those agents, such as glucagon-like peptide-1 receptor agonists and sodium–glucose co-transporter-2 inhibitors, which have been shown to reduce body weight and improve cardiovascular outcomes in these patients. Available evidence suggests that these drug classes can indeed afford a clinically meaningful BP decrease and, potentially, reduce the treatment burden. In conclusion, even though the rates of uncontrolled hypertension remain high, several novel therapeutic options are in the offing. As these emerging treatments will compound with many already available agents, future efforts should be directed at better phenotyping patients to tailor the most suitable approach for each one.

## 1. Introduction

Despite the availability of several well-tolerated and effective anti-hypertensive medications, hypertension remains the leading global risk factor for heart disease and stroke-related death and its prevalence continues to rise. According to the World Health Organization, it is estimated that more than a billion adults have hypertension worldwide, with a global prevalence of roughly one-third of the adult population [1]. Hypertension is the biggest contributor to the global burden of disease and mortality, with a yearly estimated 10 million deaths attributable to high blood pressure (BP) [2]. Yet, when applying a BP threshold of <140/90 mmHg, less than a third of hypertensive individuals have their BP controlled with anti-hypertensive drugs [3]. There is a necessity, therefore, for novel effective anti-hypertensive strategies to fill the unmet therapeutic needs of these patients.

## 2. Pathophysiology of Hypertension and General Approach to Treatment

The mechanisms underlying arterial hypertension are several and often overlapping. It is widely accepted that overactivity of the sympathetic nervous system and the renin–angiotensin–aldosterone system (RAAS) predominates in BP elevation, resulting in increased systemic vasoconstriction and salt and water retention by the kidney [4]. In addition to initiating and sustaining hypertension, overactivity of the RAAS and aldosterone excess exert deleterious cardiovascular effects independent of their BP-elevating actions, further emphasizing the importance of therapeutic interventions to ameliorate these pathophysiological states [5]. Not surprisingly, pharmacological blockade of the renin–angiotensin system is included in all major guidelines for the treatment of hypertension, which recommend, as first-line drugs, an angiotensin-converting enzyme (ACE) inhibitor or an angiotensin II type 1 receptor antagonist, a diuretic (preferably a thiazide or thiazide-like), and a long-acting calcium channel blocker. At present, steroidal mineralocorticoid receptor antagonists (MRAs) that counteract aldosterone excess, such as spironolactone or eplerenone, are considered the preferred add-on antihypertensive drugs [6].

## 3. Resistant Hypertension

In some patients, despite the use of ≥3 antihypertensive drugs of different classes, including a blocker of the renin–angiotensin system, a thiazide/thiazide-like diuretic, and a long-acting calcium channel blocker at maximal or maximally tolerated doses, BP remains uncontrolled, a condition defined as resistant hypertension (RHTN) [7]. Other recommendations for add-on therapies in these patients, in addition or alternatively to steroidal MRAs, may include amiloride, α_1_-adrenoceptor blockers, β-blockers, and centrally acting drugs (such as clonidine); all these drugs have been shown some ability to lower BP in the context of RHTN but have limited evidence for an impact on cardiovascular outcomes [7]. As a result, patients with RHTN are at greater risk of complications, such as end-stage renal disease, ischemic heart disease, heart failure, stroke, or death, compared with patients with controlled BP [8].

## 4. Novel Strategies for RAAS Targeting (Figure 1)

### 4.1. Nonsteroidal MRAs

Aldosterone, a mineralocorticoid receptor (MR) agonist released by the adrenal cortex and acting in the distal nephron, is one of the main effectors of the volume expansion in hypertension [9] and a certain degree of non-suppressible and renin-independent aldosterone production is often present in patients with RHTN [10]. This may be at least partially driven by diuretic-induced activation of the renin–angiotensin system, similar to the aldosterone ‘escape’ observed with sodium restriction or the aldosterone ‘breakthrough’ incited by pharmacological blockade of the renin–angiotensin system [11]. Notably, unopposed aldosterone-mediated BP elevation is associated with various complications, including vascular stiffening, atrial fibrillation, heart failure, ischemic heart disease, stroke, and chronic kidney disease; treatment with MR antagonists, by contrast, may reduce these adverse outcomes [12]. Meta-analyses of clinical studies on RHTN have consistently shown that steroidal MRAs (spironolactone or eplerenone) produce clinically meaningful reductions in both systolic BP and diastolic BP [13,14]. One main limitation of steroidal MRAs in clinical practice is the risk of adverse effects, particularly hyperkalemia and sex hormone-related effects [15]. To overcome these limitations, potent and highly selective non-steroidal MRAs have been developed. Finerenone, the non-steroidal MRA most extensively tested in randomized trials, has indeed a higher selectivity and binding affinity for the MR than steroidal MRAs [16]. In a couple of large, phase-3, placebo-controlled trials (FIDELIO-DKD and FIGARO-DKD), finerenone reduced both the progression of chronic kidney disease (CKD) and cardiovascular events in patients with CKD associated with type 2 diabetes (T2D) already on blockers of the renin–angiotensin system [17,18]. Consequently, finerenone has received Food and Drug Administration (FDA) and European Medicines Agency (EMA) approval for cardiorenal protection in these patients. Interestingly, the analysis in FIDELITY-TRH selected the subgroup of patients with RHTN and CKD from the FIDELITY pooled analysis of the FIDELIO-DKD and FIGARO-DKD trials [19] and indirectly compared the safety and systolic-BP-lowering efficacy of finerenone and spironolactone (the data regarding spironolactone were collected from patients with similar characteristics enrolled in the AMBER trial [20]). This comparison demonstrated that treatment with finerenone results in a lower risk of hyperkaliemia (defined as serum potassium ≥ 5.5 mmol/L) and worsening kidney function but is associated with a smaller reduction in unattended office systolic BP (−7.1 mmHg vs. approximately −11 mmHg with spironolactone). Interestingly, the placebo-adjusted decrease in systolic BP obtained with finerenone in patients of the FIDELITY-TRH analysis was greater than that seen in the overall FIDELITY population, thereby hinting at the greater anti-hypertensive potential of finerenone in patients with higher baseline BP [21]. The results of the FIDELITY-TRH analysis are in line with those of the phase-2 ARTS study, the only head-to-head clinical comparison of finerenone and spironolactone, demonstrating a smaller systolic-BP-lowering effect of finerenone than spironolactone (−4.2 mmHg vs. −10.1 mmHg, respectively) [22]. The precise reasons for this phenomenon are not entirely clear, but the very long half-life of spironolactone, in conjunction with the persistence of its active metabolites, might provide a possible explanation [23]. Despite the inferior BP-lowering effect of finerenone than traditional MRAs suggested by available data, however, its additive cardio- and renal-protective actions, as well as the lower risk of hyperkalemia, could be useful in patients of RHTN and concomitant CKD, a cohort in whom the treatment options are rather limited.

Another non-steroidal MRA with a long half-life and high receptor affinity, ocedurenone, has been tested in a double-blind, placebo-controlled, phase-3 study (CLARION-CKD) with patients with uncontrolled hypertension and advanced CKD. At the interim analysis, however, the trial failed to meet its primary endpoint (change in systolic BP from baseline to week 12) and was stopped early (https://www.novonordisk.com/news-and-media/news-and-ir-materials/news-details.html?id=168529, accessed on 18 February 2025). By contrast, the ESAX-HTN trial demonstrated that the nonsteroidal MRA esaxerenone (at doses of 2.5 or 5 mg once daily) decreases systolic BP at 4 weeks (−13.7 mmHg and −16.9 mmHg, respectively), similar to the −12.1 mmHg systolic BP change obtained with spironolactone (50 mg daily) [24]. A post hoc analysis of this trial demonstrated that esaxerenone is also effective in treating nocturnal hypertension, especially in individuals with a non-dipping pattern of night-time BP [25]. More recently, an open-label, parallel-group trial (EXCITE-HT) demonstrated that esaxerenone is non-inferior to a thiazide diuretic (trichlormethiazide) as second-line treatment in Japanese people with uncontrolled hypertension [26]. Among other possibilities, race differences in the study populations might have contributed to the discrepancy in the BP-lowering efficacy observed with different non-steroidal MRAs. Of note, exerenone has received marketing approval for the treatment of essential hypertension only in Japan [27].

**Figure 1:**
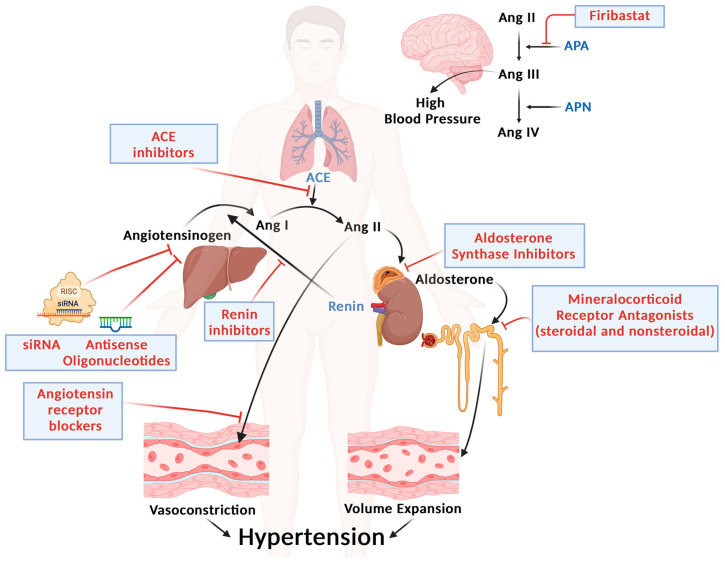
In response to various stimuli, the juxtaglomerular cells of the kidney secrete renin, which cleaves angiotensinogen into angiotensin I, which is converted to angiotensin II, primarily in the lungs, by the angiotensin-converting enzyme (ACE). Angiotensin II is a potent vasoconstrictor and stimulates cells in the zona glomerulosa of the adrenal gland to synthesize and secrete aldosterone. Aldosterone, in turn, enhances sodium reabsorption in the distal convolute tubule of the kidney. Traditional (direct renin inhibitors, ACE inhibitors, angiotensin receptor blockers) and emerging classes of drugs targeting the renin–angiotensin–aldosterone system (whose sites of action are shown here) can be used to treat hypertension. Novel drugs include nonsteroidal mineralocorticoid receptor antagonists [MRAs], aldosterone synthase inhibitors, and RNA-based therapies (small interference RNA [siRNA] and antisense oligonucleotides [ASO]) that block the synthesis of angiotensinogen in the liver. Within the brain, angiotensin II is converted by aminopeptidase A (APA) into angiotensin III, which is further converted into angiotensin IV by aminopeptidase N (APN). As brain angiotensin III has been shown to increase blood pressure, drugs have been developed (firibastat) that cross the blood–brain barrier and inhibit brain APA. Pointed arrows indicate stimulation, blunt headed arrows indicate inhibition.

### 4.2. Aldosterone Synthase Inhibitors (ASIs)

In addition to MRAs, another possible way to counteract aldosterone excess and lower BP is the use of drugs that inhibit aldosterone synthase and, consequently, decrease the production of aldosterone in the zona glomerulosa of the adrenal glands. The development of ASIs has been difficult because of the similarity between aldosterone synthase (CYP11B2) and 11β-hydroxylase (CYP11B1), the rate-limiting step in cortisol biosynthesis. Thus, earlier ASIs demonstrated a suppressive action over cortisol synthesis, which made these agents unsuitable for the treatment of hypertension. More recently, small molecule drugs that penetrate the cell membrane and selectively target aldosterone synthase were developed and tested in clinical trials [28].

In a phase-2, randomized, placebo-controlled study (Target-HTN), the ASI lorundrostat was given to adults with uncontrolled hypertension already taking two or more antihypertensive drugs. In participants with suppressed plasma renin activity (PRA), the least-squares mean placebo-adjusted difference in systolic BP following 8 weeks of treatment with lorundrostat was −9.6 mmHg for the 50 mg once-daily dose and −7.8 mmHg for the 100 mg daily dose. Similarly, 100 mg once daily of lorundrostat decreased systolic BP by −11.4 mmHg among participants without suppressed PRA [29]. The results of two ongoing phase-3 trials of lorundrostat (NCT05769608 and NCT06153693) are expected in late 2025.

In the BrigHTN trial, another ASI, baxdrostat, was shown to significantly lower BP in hypertensive patients taking at least three anti-hypertensive medications, with a least-squares mean difference between the highest dose (2 mg once daily) and placebo of −11.0 mm Hg [30]. In another study on patients with uncontrolled hypertension (HALO trial), however, baxdrostat did not significantly reduce BP compared with placebo (the changes in mean seated systolic BP for 0.5 mg, 1 mg, or 2 mg of baxdrostat or a placebo were −17.0, −16.0, −19.8, and −16.6 mmHg, respectively). A larger than anticipated placebo effect and low adherence with the study medications at some recruiting sites were evoked as possible explanations for these negative results (https://www.acc.org/Latest-in-Cardiology/Clinical-Trials/2023/03/01/23/34/halo, accessed on 18 February 2025). In light of these inconsistent data, the results of an ongoing phase-3 trial (NCT06034743) will be crucial to establish the possible role of baxdrostat as an anti-hypertensive agent.

Another phase-2, multicenter trial tested the effect of the ASI vicadrostat (BI 690517) on patients with CKD already on the maximal tolerated dose of an ACE inhibitor or an angiotensin receptor blocker [31]. It demonstrated a greater reduction in urinary albumin-to-creatinine ratio and a larger decrease in systolic BP in patients on vicadrostat than those on a placebo, which suggests renal and cardiovascular benefits. However, frequent hyperkaliemia, leading at times to treatment discontinuation, and some cases of adrenal insufficiency raised concerns about the possible risks associated with this drug. Larger trials would be needed to ultimately show the efficacy and safety of this approach as a treatment option for these patients.

### 4.3. Inhibition of Angiotensinogen

While hypertension is responsible for serious cardiovascular and cerebrovascular complications, it is referred to as a ‘silent killer’ because the affected individuals often remain asymptomatic for many years. It is not surprising, therefore, that asymptomatic patients have little motivation to seek and adhere to treatment, which typically requires daily oral administration of one or more hypotensive agents [32]. This aspect is even more relevant in patients with RHTN, in whom multi-pill regimens often lead to medication non-adherence, one of the commonest causes of pseudo-resistance [33]. Transforming a daily multi-pill treatment into a single subcutaneous injection to block, for weeks or even months, the hepatic production of a protein critically involved in hypertension is, therefore, considered an efficient BP-lowering strategy to overcome non-adherence.

As the most upstream component of the renin–angiotensin–aldosterone system, angiotensinogen represents an attractive target for lowering BP. It is primarily produced in the liver, making it ideal for RNA-based therapies that genetically inhibit the RAAS through the reduced synthesis of its precursor. Zilebesiran is an angiotensinogen-targeting small interfering RNA (siRNA) linked to an N-acetylgalactosamine ligand to bind with high affinity to the hepatic asialoglycoprotein receptor [34]. Because of its effectiveness in reducing hepatic angiotensinogen mRNA by inhibiting its synthesis, zilebesiran was recently tested in clinical trials. In a double-blind, placebo-controlled, phase-2 study (KARDIA-1), patients with mild-to-moderate hypertension, following a washout period of 2 to 4 weeks from the previous antihypertensive medications, were randomized to receive either subcutaneous zilebesiran (at different doses every 3 or 6 months) or a placebo. Patients receiving zilebesiran had a significant decrease in mean ambulatory systolic BP, which was consistent over 24 h and sustained for up to 6 months [35]. In another phase-2 trial (KARDIA-2), after discontinuing previous antihypertensive medications, hypertensive patients were randomized to take one of three once-daily medications: indapamide, amlodipine, or olmesartan. After at least four weeks, all patients underwent 24 h BP monitoring. Those who consistently took their medications and still had an elevated average 24 h systolic BP (between 130 and 160 mmHg) were randomized to receive a single injection of either 600 mg of zilebesiran or a placebo. At the follow-up 6 months later, an additional reduction in 24 h ambulatory systolic BP was observed with zilebesiran compared to the placebo, on top of any other drug (https://www.jacc.org/doi/abs/10.1016/S0735-1097%2824%2903659-3, accessed on 18 February 2025). Another ongoing trial (KARDIA-3) is testing the efficacy and safety of zilebesiran in patients with high cardiovascular risk or advanced chronic kidney disease, with hypertension uncontrolled on two to four BP medications (NCT06272487).

Antisense oligonucleotides (ASOs) that prevent, through disparate mechanisms, the translation of messenger RNA into protein are another possible approach for lowering angiotensinogen. IONIS-AGT-L_Rx_ is an ASO that reduces the synthesis of angiotensinogen in the liver by increasing the degradation of its mRNA [36]. In a couple of small, phase-2 clinical studies in hypertensive patients, IONIS-AGT-L_Rx_ was administered by weekly subcutaneous injections, either as a monotherapy in patients with controlled BP (NCT03714776) or as an add-on therapy in patients with uncontrolled hypertension (NCT04083222). A significant reduction in circulating angiotensinogen levels (the primary endpoint) was observed after IONIS-AGT-L_Rx_ in both studies. The trials, however, were not powered to detect differences in BP, and the observed post-treatment decrease in BP was not significantly different between patients receiving the active drug or the placebo [37]. The results of another phase-2 study with IONIS-AGT-L_Rx_ (ASTRAAS trial, NCT04714320) and those of a phase-2 study with ION-904 (tonlamarsen; NCT04731623), a next-generation ASO enabling less frequent (once monthly) dosing, in patients with uncontrolled hypertension have not yet been released.

Finally, studies in animal models of hypertension have shown that targeting angiotensinogen by CRISPR-Cas9 gene editing results in a sustained BP reduction [38]. These findings have fostered hope that a once-in-a-lifetime treatment could lead to a definitive cure for hypertension [39], but still need to be verified in clinical studies.

### 4.4. Aminopeptidase A (APA) Inhibitors

APA is the enzyme that cleaves angiotensin II into angiotensin III (Ang III), a peptide that, in the brain, promotes the release of vasopressin and enhances sympathetic drive, thereby leading to high BP [40]. Earlier studies in experimental models demonstrated that brain but not systemic Ang III is responsible for BP control, given that APA inhibitors not crossing the blood–brain barrier do not affect systemic BP; APA inhibitors that cross the blood–brain barrier, by contrast, induce a dose-dependent BP reduction [41]. Firibastat is an orally active inhibitor of brain APA that has been tested as a possible treatment for human hypertension. In an open-label, phase-2 trial, the administration of firibastat to patients with uncontrolled hypertension resulted in a significant reduction in automated office systolic BP (−9.5 mmHg) and diastolic BP (−4.2 mmHg) [42]. In a subsequent phase-3, multicenter, placebo-controlled trial (FRESH), however, firibastat (500 mg twice daily) did not confirm a significant BP-lowering effect but induced frequent allergic skin reactions (https://sessions.hub.heart.org/aha-22/article/22539144/firstinclass-aminopeptidasea-inhibitor-fails-to-reduce-treatmentresistant-hypertension, accessed on 18 February 2025). Because of these results, a concomitant phase-3 clinical trial (REFRESH), testing firibastat in patients with CKD was stopped and further development of the drug has been suspended.

## 5. Other Drug Classes

### 5.1. Endothelin Receptor Antagonists (ERAs) (Figure 2, Panel A)

Because endothelin (ET)-1 is the most potent natural vasoconstrictor, arterial hypertension is one of the first clinical conditions for which endothelin receptor antagonists (ERA) have been investigated. Some studies have indeed shown the efficacy of ERA in lowering BP [43]; still, the availability of alternative antihypertensive drugs with better tolerability and safety profiles has limited the use of these drugs for the management of hypertension. Nonetheless, in a significant number of hypertensive individuals whose BP remains uncontrolled or resistant to combination therapy, activation of the ET-1 system plays a crucial pathogenic role [44]. This is particularly evident in those cases of resistant hypertension related to obesity, where the up-regulation of ET-1 signaling within the vascular wall contributes to the rise of systemic vascular resistance [45]. Earlier studies assessing the effectiveness of the selective ET_A_ receptor antagonist darusentan in patients with RHTN have shown inconsistent outcomes when office or 24 h ambulatory blood pressure were employed as a treatment endpoint [46,47]. Thus, although darusentan was superior to a placebo in reducing mean 24 h BP, it did not show similar efficacy on office BP, largely due to an unexpectedly greater reduction in sitting BP in the placebo group [47]. Additionally, darusentan was associated with a high incidence of fluid retention/edema, leading to greater treatment discontinuation rates than the placebo due to adverse events. Consequently, this treatment approach was stopped for several years. However, a recent multicenter, randomized, phase-3 trial (PRECISION) has reignited the interest in ERA for the treatment of RHTN [48]. This trial demonstrated that aprocitentan, an active metabolite of macitentan that blocks both ET_A_ and ET_B_ receptors, results in a clinically meaningful reduction in both office and 24 h ambulatory BP in patients with RHTN [48]. Based on these findings, aprocitentan has recently received approval from the FDA for the management of RHTN in the United States. The tolerability and safety profiles of aprocitentan, along with its potential influence on adherence to multidrug regimens used in RHTN, still represent an important concern [7]. In the PRECISION trial, the adverse events related to aprocitentan (primarily due to symptomatic fluid retention) were not negligible, particularly at the highest drug dose. This aspect warrants caution in patients at a higher risk of fluid overload (such as those with diabetes, chronic kidney disease [CKD], or a history of heart failure) and emphasizes the need for further studies that use ERA at the lowest effective dose while enhancing diuretic treatment in those patients with RHTN that are more prone to fluid retention.

### 5.2. Glucagon-like Peptide-1 Receptor Agonist (GLP-1 RA)-Based Therapies (Figure 2, Panel B)

Obesity is one of the most important modifiable risk factors for RHTN as a high percentage of individuals with RHTN are overweight or obese [49]. The mechanisms linking overweight and obesity with RHTN are multiple and not fully characterized. Increased activity of the RAAS, hyperaldosteronism (even independent of activation of the RAAS), insulin resistance, increased sympathetic activity, oxidative stress, and systemic inflammation, typically seen in individuals with visceral adiposity, are all processes potentially contributing to RHTN in these patients [50].

Glucagon-like peptide-1 receptor agonists (GLP-1 RAs) are anti-obesity drugs that were recently shown to be effective in reducing body weight and ameliorating cardiovascular outcomes in patients with overweight or obesity [51]. As GLP-1 RAs have also demonstrated a certain degree of BP-lowering effect, several mechanisms have been evoked to explain it, including actions on the nervous system to counteract sympathetic hyperactivity, on the kidney to stimulate natriuresis, and on the vasculature to promote vasodilation [52]. Among the first GLP-1-based treatments approved for weight management in obese patients, liraglutide and semaglutide have both demonstrated mild BP-lowering actions in randomized clinical trials. Thus, a meta-analysis of 12 such trials has shown that treatment with liraglutide (3 mg once daily) decreases both systolic BP (−3.1 mmHg) and diastolic BP (−1.0 mmHg) compared to a placebo [53]. Similarly, a recent meta-analysis demonstrated that once-weekly semaglutide results in a mean difference of −3.4 mmHg in systolic BP and −0.7 in diastolic BP compared to a placebo [54].

**Figure 2:**
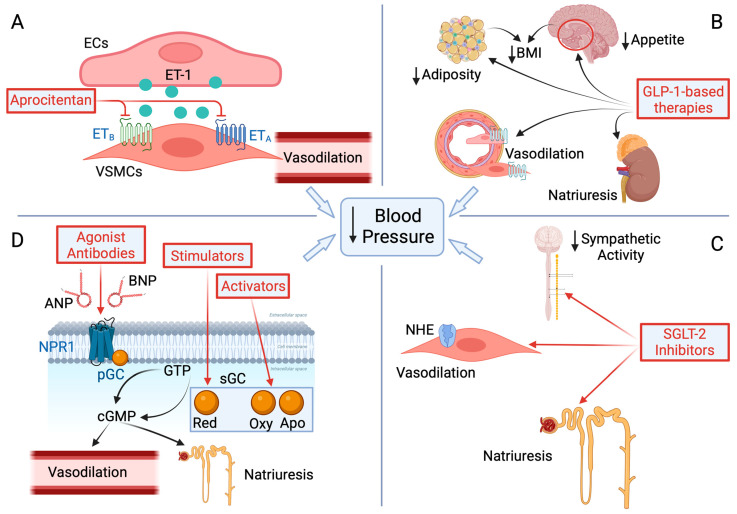
Among drugs not directly acting on the renin–angiotensin–aldosterone system that have been shown to possess blood pressure-lowering effects are the dual endothelin receptor antagonist aprocitentan (**A**), the anti-diabetic and anti-obesity drug class of glucagon-like peptide-1 (GLP-1) receptor agonists (**B**), the anti-diabetic sodium–glucose cotransporter 2 (SGLT-2) inhibitors (**C**), and drugs acting on the guanylate cyclase–cyclic guanosine monophosphate (cGMP) system, such as agonist antibodies to natriuretic peptide receptor 1 (NPR1) and stimulators or activators of soluble guanylate cyclase (sGC) (**D**). ECs, endothelial cells; VCSMs, vascular smooth muscle cells; ET-1, endothelin-1; ET_A_, type A endothelin receptor; ET_B_, type B endothelin receptor; BMI, body mass index; NHE, sodium–hydrogen exchanger; ANP, atrial natriuretic peptide; BNP, B-type natriuretic peptide; pGC, membrane-bound guanylate cyclase; GTP, guanosine triphosphate; Red, reduced isoenzyme; Oxy, oxidized isoenzyme; Apo, apo isoenzyme. Pointed arrows indicate stimulation, blunt headed arrows indicate inhibition.

Tirzepatide is a dual GLP-1 and glucose-dependent insulinotropic polypeptide (GIP) receptor agonist that has synergic effects on reducing appetite, increasing metabolic function, and decreasing food intake [55]. Interestingly, this combination has demonstrated a more significant impact on reducing body weight and BP compared to GLP-1 RAs alone. Thus, in the SURMONT-1 trial, once-weekly tirzepatide (5, 10, or 15 mg) resulted in marked, dose-dependent weight loss (up to 20%) associated with a reduction in office diastolic BP (−4.0 mmHg) compared to a placebo [55]. More recently, the SUMMIT trial tested the effects of tirzepatide (up to 15 mg subcutaneously once weekly for at least 52 weeks) in obese patients (BMI ≥ 30 kg/m^2^) with heart failure with preserved ejection fraction (≥50%); in addition to lowering the risk of a composite of death from cardiovascular causes or worsening heart failure, tirzepatide resulted in a significant placebo-adjusted decrease in systolic BP (−4.7 mmHg) [56]. Excess adiposity is also an etiologic risk factor for obstructive sleep apnea, a breathing disturbance during sleep associated, through various mechanisms, with increased night-time BP and major cardiovascular complications [57]. Interestingly, in a recent phase-3, double-blind, placebo-controlled trial (SURMOUNT OSA), adults with obesity and moderate-to-severe obstructive sleep apnea were assigned to receive either the maximum tolerated dose of tirzepatide (10 or 15 mg) or a placebo for 52 weeks. Significant improvement from baseline in the measurement of the apnea–hypopnea index (the number of apneas and hypopneas during an hour of sleep) was observed in participants receiving tirzepatide. In addition, the estimated tirzepatide vs. placebo treatment difference at week 48 was −7.8 mmHg for systolic BP and −2.8 mmHg for diastolic BP [58]. As home BP or 24 h ambulatory BP have been demonstrated to be better independent predictors over office BP of cardiovascular events and treatment-induced reductions of overall cardiovascular risk [59], it is recommended to incorporate out-of-office BP monitoring in trials testing the effectiveness of anti-hypertensive drugs. In line with these recommendations, a subgroup of patients participating in the SURMONT-1 trial, whose BP was already controlled (≤140/90 mmHg) by stable (≥3 months) anti-hypertensive treatment, underwent 24 h ambulatory BP monitoring at baseline and week 36 of treatment. The placebo-adjusted systolic BP change from baseline was −7.4 mmHg for 5 mg of tirzepatide, −10.6 mmHg for 10 mg of tirzepatide, and −8.0 mmHg for 15 mg of tirzepatide; the results were consistent for both day- and night-time systolic BP, with significant reductions in systolic BP vs. the placebo for each tirzepatide dose. At week 36, 24 h diastolic BP was significantly lower from baseline vs. the placebo in patients who received tirzepatide at doses of 5 mg (−2.0 mmHg) and 10 mg (−2.9 mmHg), but not in the 15 mg group (−0.5 mmHg) [60].

Other GLP-1-based treatments, although not commercially available, have demonstrated remarkable weight-reducing and BP-lowering effects in overweight and obese individuals. Thus, in a phase-2 clinical trial including adults without diabetes but with overweight or obesity plus ≥ 1 weight-related coexisting condition (hypertension, dyslipidemia, obstructive sleep apnea, or cardiovascular disease), retatrutide, a synthetic triple GLP-1, GIP, and a glucagone receptor agonist resulted in a significant reduction of systolic BP (−12.1 mmHg) and diastolic BP (−8.1 mmHg) at 48 weeks [61]. Strikingly, retatrutide treatment also led to the discontinuation of at least 1 antihypertensive medication. This suggests that even though no study has directly assessed the impact of GLP-1-based therapies on RHTN, this class of drugs might reduce the treatment burden in these patients, improving both BP control and medication adherence. Similar results were observed in a phase-2 placebo-controlled trial in nondiabetic patients with obesity (BMI ≥ 30 kg/m^2^) or overweight (≥27 kg/m^2^) plus at least 1 weight-related condition that assessed the effects of four doses of an orally active, non-peptide small molecule (orfoglipon) compared to a placebo. In addition to weight reduction, treatment with orfoglipon was associated with an impressive decrease in systolic BP (−10.1 mmHg at the highest dose), considerably higher than that observed with the placebo (−1.8 mmHg) [62]. Finally, another phase-2 trial in patients with T2D and a BMI ≥ 27 kg/m^2^ treated with once-weekly CagriSema, a combination of semaglutide and the long-acting amylin analog cagrilintide, demonstrated greater weight loss and BP decrease at 32 weeks in patients treated with the combination of the two substances (SBP reduction by >10 mmHg) than in those treated with either semaglutide or cagrilintide alone [63].

Taken collectively, these findings suggest that GLP-1-based therapies, in addition to sustained weight loss and an overall reduction of cardiovascular risk, can afford an anti-hypertensive effect [64]. Given the expanding availability of new drugs in this class and the strong motivation of patients to take them, they should certainly be considered for the treatment of patients with increased BMI and high BP, especially those with RHTN or other co-morbidities. It must be considered, however, that these drugs have frequent side effects, especially on the gastrointestinal tract, and that their high cost may limit their use, especially in those low-income populations that are at a higher risk of obesity-related hypertension. Also, the use of these medications should integrate, rather than substitute, those general measures, such as diet and physical activity, that remain the mainstay management of overweight and obesity.

### 5.3. Sodium–Glucose Cotransporter-2 (SGLT-2) Inhibitors (Figure 2, Panel C)

SGLT-2 inhibitors, originally used for improving glycemic control in T2D, have been consistently shown to reduce the risk of adverse cardiovascular events, worsening heart failure, and chronic kidney disease progression in both diabetic and non-diabetic patients [65]. Even though SGLT-2 inhibitors are not classified as antihypertensive agents, they have also been shown to lower BP through a combination of effects, including natriuresis, vasodilation, and lower sympathetic nerve activity [66].

An analysis of available data on changes in office or out-of-office BP during treatment with SGLT-2 inhibitors made by Kario et al. evidenced consistent reductions in home, 24 h, daytime, and nocturnal BP during treatment with SGLT-2 inhibitors in patients with diabetes and hypertension; patient subgroups with a higher BMI and higher baseline BP had greater reductions in BP during treatment with SGLT-2 inhibitors [67,68]. A post hoc analysis of the DELIVER trial evaluated the effects of dapagliflozin among participants with apparent treatment-resistant hypertension (aTRH). To this end, DELIVER participants were categorized based on baseline BP, with aTRH defined as BP ≥ 140/90 mmHg (≥130/80 mmHg if diabetics) despite treatment with three antihypertensive drugs including a diuretic. Non-resistant hypertension was defined as BP above the threshold but not meeting the aTRH criteria. Participants with aTRH exhibited the greatest absolute reduction in the rate of primary events with dapagliflozin compared with non-resistant hypertension and controlled BP. Dapagliflozin modestly reduced systolic BP (by approximately 1 to 3 mmHg), without increasing the risk of hypotension, hypovolemia, or other serious adverse events, but did not improve the proportion of participants with aTRH attaining goal BP over time [69]. A recent, retrospective cohort study of approximately 13,000 adults who were already using drug therapy for hypertension examined the effect of SGLT-2 inhibitors, primarily prescribed for the management of diabetes, on BP and the use of antihypertensive medication. The authors reported that the average reduction in systolic BP and diastolic BP was 5.3 mmHg and 2.5 mmHg, respectively, and the BP decrease was slightly greater in patients with RHTN. Thirteen percent more patients achieved a BP goal of <130/80 mmHg after starting SGLT-2 inhibitors and over one-third of all patients used fewer antihypertensive medications after they were started on SGLT-2 inhibitors [70].

At odds with the notion that the BP-lowering effect of SGLT-2 inhibitors is greater in patients with higher BP or RHTN, however, are the results of a post hoc analysis of the CREDENCE trial. It examined the office-BP-lowering effect of canagliflozin (100 mg once daily) overall and in subgroups defined based on baseline systolic BP, number of BP-lowering drug classes, and history of aTRH. Canagliflozin recipients showed an early and sustained reduction in BP (–3.5 mm Hg versus baseline), but, surprisingly, there was no apparent BP-lowering effect of the drug in patients with the highest baseline BP [71]. Similarly, a recent study by Tsukamoto et al. retrospectively evaluated Japanese patients with T2D and chronic kidney disease (CKD) treated with SGLT-2 inhibitors for at least 1 year. The primary outcome was achieving the target BP (<130/80 mmHg) after SGLT-2 inhibitor treatment. The authors reported that the BMI < 29.1 group had significantly lower systolic and diastolic BP values after SGLT-2 inhibitor treatment than the BMI ≥ 29.1 group, thereby hinting that the BP-lowering effect of SGLT-2 inhibitors is greater in non-obese patients [72]. The precise reasons for these counterintuitive findings are unclear, but the age and race characteristics of the study populations, as well as the concurrent anti-hypertensive regimens, may provide a potential explanation [68].

Finally, it has been speculated that the magnitude of BP reduction afforded by SGLT-2 inhibitors appears similar to that seen with other recently FDA-approved treatments for patients with hypertension, such as aprocitentan [48] and renal denervation [73]. It seems worth considering, therefore, SGLT-2 inhibitors as add-on therapy for patients with diabetes and hypertension, particularly those with RHTN. Future studies, however, are needed to better identify the predictors of the BP-lowering response to SGLT2 inhibitors. It will also be important to ascertain the optimal treatment targets and the most suitable BP-lowering strategy (ideally by head-to-head comparison of SGLT-2 inhibitors and the newest GLP-1-based therapies) in a sizeable, high-risk group of hypertensive patients with obesity and diabetes.

### 5.4. Natriuretic Peptide Receptor 1 (NPR1) and Soluble Guanylate Cyclase (sGC) Agonists (Figure 2, Panel D)

Natriuretic peptides regulate the vascular tone through the natriuretic peptide receptor 1 (NPR1). Thus, their binding with NPR1 results in the activation of membrane-bound guanylate cyclase (GC) and the synthesis of cyclic guanosine monophosphate (cGMP), the second messenger for a downstream signaling cascade that drives a variety of beneficial cardiorenal effects, including natriuresis, and vasodilation. Not surprisingly, NPR1 has recently become a target of long-acting activators aimed at exploiting its BP-lowering actions.

XXB750 is a fully human monoclonal antibody designed to specifically activate the NPR1. In a first-in-human clinical study (NCT06142383) including 73 healthy participants, XXB750 significantly increased plasma cGMP levels and reduced systolic BP during ambulatory BP monitoring. Importantly, its systolic-BP-lowering effect was maintained at day 28; even though its effect on diastolic BP was less pronounced, this sustained anti-hypertensive action mandates further studies to verify whether XXB750 once monthly has the potential for future development as a novel therapy for cardiovascular conditions (https://www.ahajournals.org/doi/epub/10.1161/CIR.0000000000001200, accessed on 18 February 2025).

REGN5381 is another NPR1 agonist monoclonal antibody that has shown sustained hemodynamic effects in animal models [74] and is in early clinical trials in humans. A recent double-blind, single-ascending dose study (NCT04506645) randomized 24 mildly hypertensive patients to receive a single intravenous administration of three REGN5381 dose levels or a placebo. In participants treated with the highest dose of REGN5381, maximum decreases in systolic BP of −18 mmHg and diastolic BP of −8 mmHg occurred within 24 h post-dose and were sustained for over 72 h (the full duration of inpatient monitoring), thereby suggesting prolonged hemodynamic effects (https://www.jacc.org/doi/epdf/10.1016/S0735-1097%2824%2903696-9, accessed on 18 February 2025).

Soluble GC (sGC) is a key enzyme responsible for vasodilation and, as such, it has attracted growing interest as a possible therapeutic target in various cardiovascular diseases. Two classes of sGC agonists are available: stimulators (which increase the enzymatic activity of the reduced form of sGC) and activators (which enhance the activity of the oxidized and apo forms of sCG), which have shown antihypertensive effects in preclinical models of hypertension [75]. In addition, various phase-1 trials have provided preliminary evidence that sCG agonists may exert a BP-lowering effect in humans. Thus, a study testing IW-1973, a sCG stimulator given daily for 2 weeks to patients with T2D and hypertension on a stable anti-hypertensive regimen, demonstrated a greater reduction of 24 h ambulatory BP with IW-1973 than a placebo, especially in patients with inadequate BP control (NCT03091920). Another sCG stimulator (BAY 1021189) is in a clinical trial with patients with metabolic syndrome (NCT05711719) and a pair of sCG activators (BAY 1101042 and BAY 3283142) are in clinical trials with patients with chronic kidney disease or hypertension (NCT04507061 and NCT6428825, respectively) [76].

## 6. Future Perspectives

Even though the rates of uncontrolled hypertension remain unacceptably high, several novel therapeutic options are in the offing. Because these emerging treatments will compound with many anti-hypertensive agents already available, future efforts should be mainly directed at better identifying which patients might have a better BP response to one treatment over another. Thus, better phenotyping may assist healthcare providers in tailoring the most suitable approach for each patient. For instance, individuals with hypertension linked to dysregulated aldosterone may benefit most from treatment with an MRA or an ASI, while those affected by obesity- or diabetes-associated hypertension might be better managed with a GLP-1 RA or SGLT-2 inhibitor. This targeted approach might result in a reduction in the number of daily pills that patients, especially those with difficult-to-control hypertension, should take, thereby reducing non-adherence to treatment. The availability of drugs with weekly or monthly administration rates will be of further help in this regard. Nevertheless, additional research is required before implementing these strategies in clinical practice. The growing research and awareness surrounding new therapies for the effective management of hypertension is a positive trend that will hopefully encourage both patients and healthcare providers to address uncontrolled hypertension with greater attention.

## Data Availability

Not applicable.
